# A Time–Frequency Acoustic Emission-Based Technique to Assess Workpiece Surface Quality in Ceramic Grinding with PZT Transducer

**DOI:** 10.3390/s19183913

**Published:** 2019-09-11

**Authors:** Martin A. Aulestia Viera, Paulo R. Aguiar, Pedro Oliveira Junior, Felipe A. Alexandre, Wenderson N. Lopes, Eduardo C. Bianchi, Rosemar Batista da Silva, Doriana D’addona, Andre Andreoli

**Affiliations:** 1 Department of Electrical Engineering, São Paulo State University—UNESP, Av. Eng. Luiz Edmundo Carrijo Coube, 14-01, Bauru 17033-360, Brazil; 2 Department of Mechanical Engineering, São Paulo State University—UNESP, Av. Eng. Luiz Edmundo Carrijo Coube, 14-01, Bauru 17033-360, Brazil; 3 School of Mechanical Engineering, Federal University of Uberlandia, Av. João Naves de Avila 2121, Uberlandia 38408-100, Brazil; 4 Fraunhofer Joint Laboratory of Excellence on Advanced Production Technology (Fh-J_LEAPT Naples) Department of Chemical, Material and Industrial Production Engineering, University of Naples Federico II, Piazzale Tecchio 80, 80125 Naples, Italy

**Keywords:** piezoelectric transducer, sensor monitoring, ceramic grinding, digital signal processing, acoustic emission, short-time Fourier transform

## Abstract

Innovative monitoring systems based on sensor signals have emerged in recent years in view of their potential for diagnosing machining process conditions. In this context, preliminary applications of fast-response and low-cost piezoelectric diaphragms (PZT) have recently emerged in the grinding monitoring field. However, there is a lack of application regarding the grinding of ceramic materials. Thus, this work presents an analysis of the feasibility of using the acoustic emission signals obtained through the PZT diaphragm, together with digital signal processing in the time–frequency domain, in the monitoring of the surface quality of ceramic components during the surface grinding process. For comparative purpose, an acoustic emission (AE) sensor, commonly used in industry, was used as a baseline. The results obtained by the PZT diaphragm were similar to the results obtained using the AE sensor. The time–frequency analysis allowed to identify irregularities throughout the monitored process.

## 1. Introduction

Grinding is a precision abrasive machining process used extensively for producing components with fine tolerances and high surface quality [1]. Among all the variables measured during the grinding process, the surface integrity is one of the most important parameters of any machined surface and is a decisive factor in the evaluation of a successful grinding, especially when high surface integrity requirements need to be met for some applications [2]. The monitoring of the grinding process is very complex because of the high number of influencing parameters, such as the workpiece, the grinding machine, and the process parameters. The control and monitoring of the grinding process allow the improvement of the process performance and the reduction of defects to a possible minimum to guarantee high precision and quality [3,4].

According to Teti et al. [5], the monitoring of machining operations has traditionally been categorized into two approaches: Direct and indirect. In the direct approach, the actual quantity of the variable, e.g., tool wear, is measured. On the other hand, the indirect approach uses auxiliary quantities, e.g., sensor signals (acoustic emission (AE), power, vibration, and force), to estimate a variable quantity. According to [6], among all the signals of the mentioned sensors, the acoustic emission (AE) is considered the most sensitive signal because its frequency range is beyond mechanical vibrations and electrical noises and, therefore, these noises can be easily filtered. According to Lopes et al. [7], the acoustic emission signal is defined as the transient elastic waves generated by the rapid release of energy within a material. Regarding this subject, AE techniques have been widely employed to monitor engineering applications [8,9,10,11,12]. For example, in He et al. [13], the AE method for detecting metallic grinding burn was presented as a nondestructive detection method. Lopes et al. [14] studied the influence of the temperature on the frequency response of an AE sensor. In Badger et al. [15], the fundamental relationships between the AE signal and the dressing variables were proposed.

Recently, piezoelectric diaphragms of lead zirconate titanate (PZT) have become popular due to the fact that it has been successfully used in many scientific applications in view of its low cost and excellent sensing capacities [16,17]. The PZT diaphragms have a simple construction consisting of a brass plate on which a ceramic disc is fixed. In the past, these acoustic components were only used for sound generation (buzzers and telephone receivers). However, the considerable potential of these transducers makes them attractive to other applications besides producing sound [18]. In this context, PZT diaphragms have been successfully used in the areas of structural health monitoring (SHM) to detect structural damages in engineering projects by signals related to the piezoelectric effect [19]. In addition, they have also been used as ultrasonic actuators [20], as the active element in acoustic position encoder [21], and as an acoustic sensor for partial discharge monitoring in power transformers [22].

The present work differs from other studies because it proposes a non-invasive and low-cost monitoring technique of the ceramic grinding process through the implementation of the PZT diaphragm and digital signal processing in the time–frequency domain, which allows a better interpretation of the results when compared to the traditional techniques. An important aspect of this research work is the use of the PZT diaphragm, which is a low-cost sensor compared to sensors such as acoustic emission, dynamometer, accelerometer, and power. PZT diaphragms can operate in both active and passive configurations and have an average cost of a few cents versus the high average cost of AE sensors, which range from hundreds to thousands of dollars [23]. In addition, they are compact, flexible, lightweight, and simple acoustic components widely used in various electronic devices to produce sound (alarm, ringing, and beep) [18,24]. Some preliminary applications of the PZT diaphragm emerged in the grinding field focusing on workpiece surface integrity and tool condition monitoring. For example, in Batista da Silva et al.’s study [25], the electromechanical impedance (EMI) method with low-cost PZT diaphragms were used to monitor the surface damage of ground steel workpieces. Microhardness and roughness measurements were compared with the results of the proposed technique. In Marchi et al. [26], two PZT diaphragms were used to detect the workpiece wear by means of the EMI method. The workpiece wear was correlated to the calculated damage statistics. Ribeiro et al. [27] proposed a new technique for monitoring the surface burning on steel workpieces using the PZT diaphragm and two types of grinding wheels (cubic boron nitride and aluminum oxide). An acoustic emission sensor was used to verify the efficiency of the PZT diaphragm. In Junior et al. [28], an approach for monitoring the dressing operation by means of PZT diaphragm-based impedance was proposed. The authors validated the proposed approach based on artificial neural networks (ANN), which selected the most damage-sensitive features based on the optimal frequency band.

The present work is an expansion of the research activities described in Viera et al. [16,29], where initial results were presented. Therefore, the scope of this research work is to illustrate a new broader approach for the real-time monitoring system of the surface quality of ground ceramics using the low-cost PZT diaphragm. In this context, the results of the time–frequency analysis, obtained through the short-time Fourier transform (STFT), are used as the basis for the computation of the ratio of power (ROP) metric, which is considered an important metric in the frequency domain. However, in the present work, the ROP was computed in the time domain based on the study of Thomazella et al. [30], who conducted a pioneer work with the time–frequency approach along with the time domain ROP for monitoring self-vibrations on ground steel workpieces. The application of the technique in the monitoring of the ceramic grinding process in order to estimate the workpiece surface quality represents a novel approach for the manufacturing field. Moreover, this work differs from [30] because of the use of the low-cost piezoelectric diaphragm instead of the accelerometer. In addition, in order to verify the effectiveness and reliability of the proposed approach, an acoustic emission (AE) sensor, consolidated in the monitoring of manufacturing processes, was used. This paper is organized as follows: A ceramic grinding overview is presented in Section 2. Section 3 and Section 4 describe the piezoelectric diaphragms and the signal processing techniques used in this study. The experimental setup is shown in Section 5. Section 6 presents the results and discussion. Finally, the conclusions are presented in Section 7.

## 2. Ceramic Grinding Overview

Ceramic components are susceptible to damages, such as cracks and residual stress due to their extreme hardness and high brittleness, which may affect the surface properties of the material [31]. Ceramic machining using diamond cutting tools is the primary technique for achieving specified dimensions and acceptable surface finish. Depending on the application of the component, the machining of the synthesized ceramics can represent more than 50% of the production cost, compared to 5% to 15% for metallic components. Among all machining processes, grinding represents more than 80% of all ceramic machining [32]. According to Brinksmeier et al. [33], the results of a grinding process can be subdivided into characteristics concerning the geometry and surface integrity of the workpiece. The essential macro geometric characteristics are dimension, shape, and waviness, while surface roughness is the main micro geometric characteristic. The surface integrity can be described by residual stresses, hardness, and material structure.

In Nascimento et al. [34], the viability of ceramic grinding with minimum quantity lubrication (MQL) with water was studied. A total of 45 grinding tests were performed with different MQL water concentration, depth of cut, and feed rate. The measured output variables were surface roughness, power, and scanning electron micrographs. The results show that MQL water–oil (1:1) was superior to conventional lubrication in terms of surface quality. Thomas et al. [35] proposed a new mathematical model to predict the surface roughness of ground ceramics. The effectiveness of this model was proved by the comparison of the experimental results with the predicted results. The author concluded that the optimization of the surface roughness can be done by controlling the grinding parameters. Liu et al. [36] studied the effects of the grinding parameters in the silicon nitride ceramic grinding. The influence of the grinding parameters, such as grain size, wheel speed, workpiece speed, and grinding depth, were analyzed regarding their effects on the grinding force, surface roughness, and subsurface damage. The ceramic grinding process can be optimized by the correct choice of grinding parameters. It is worth mentioning that the cited works used the surface roughness as the main variable in the evaluation of the quality of the workpiece surface.

The workpiece surface roughness is usually the most significant evaluation indicator in assessing the quality of ground surfaces. The evaluation of the competitiveness of the system can be performed through the estimation of the surface roughness, allowing productivity improvements and reducing costs [37]. The mean surface roughness (*R_a_*) is defined as the arithmetic average of the absolute values of the deviations of the surface profile height from the mean line within the sampling length *l* [38]. Another tool used to evaluate the surface characteristics is the confocal microscopy. Thus, surface changes can be evaluated by reconstructing the topography of the surface from optical sections and light reflection [39]. In the ceramic grinding process, confocal microscopy can be found in the study of the cutting tool modeling [40] and the estimation of the maximum depth of cut during MQL grinding [41].

The monitoring of the ceramic grinding process by sensors has barely been studied, and only a few studies have been published. Nakai et al. [42] performed the estimation of the surface roughness of ceramic components through artificial neural networks (ANN). Four artificial models were tested with input statistics derived from the AE and power signals. The results obtained an accuracy of over 90%, which contributes to the automation of the ceramic grinding process. Feng et al. [43] monitored the cutting tool wear during ceramic micro-end grinding using AE, vibration, and force signals. The feasibility of monitoring the wear of the ceramic micro-grinding tool without knowing the machining characteristics was verified. Junior et al. [44,45] performed a feature extraction using the frequency domain spectrum and time-domain analysis of vibration signals to monitor the advanced ceramic grinding process. The statistics calculated by the application of digital filters in the chosen frequency bands were correlated with the workpiece surface roughness. Finally, the statistical analysis indicated similarity and confidence among the results presented.

## 3. Piezoelectric Diaphragms

**Figure 1:**
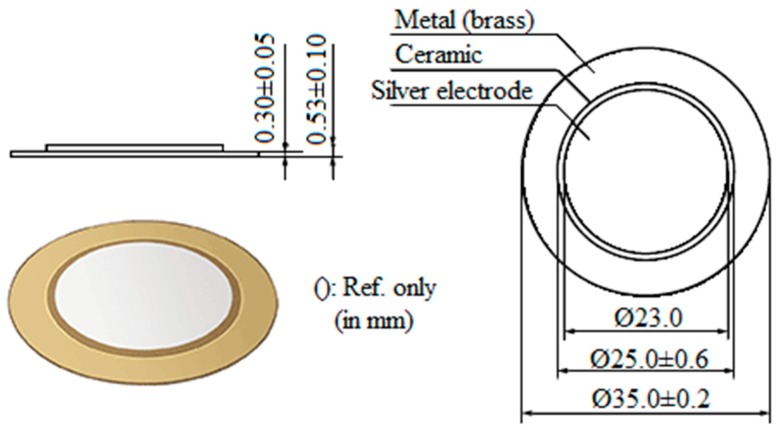
Murata piezoelectric sensor 7BB-35-3 used in the tests.

The piezoelectric diaphragms have a very simple construction and are available in different sizes. This type of transducer consists of a circular piezoelectric ceramic (active element typically of barium titanate or PZT), usually ranging from 0.1 to 2 mm in thickness, mounted on a circular metal plate (diaphragm available in brass, nickel alloy, or stainless steel). The ceramic is coated with a thin metallic film (usually silver) that serves as an electrode. Figure 1 shows a typical PZT diaphragm and its parts [18]. Piezoelectric transducers can operate both as sensors and actuators due to the piezoelectric effect [46]. The piezoelectric effect consists of the generation of an electric dipole in a material that is subjected to a mechanical force, which results in an electrical output voltage. The polarization produced by the voltage creates charges and therefore an electric field. In the reverse effect, the application of an electric voltage in the piezoelectric material causes a mechanical deformation [47].

According to Castro et al. [22], in piezoelectric material, there is an electromechanical coupling, i.e., an electric field applied to the material generates a mechanical deformation while a mechanical change generates an electric load. Thus, piezoelectric transducers are capable of generating an electrical voltage when altered by a mechanical stress, which is generated by an acoustic wave. The application of piezoelectric sensors for different purposes can be found in the specific literature [48,49].

## 4. Signal Processing

The discrete Fourier transform (DFT) is a method for the analysis of frequency spectra in digital signal processing, usually implemented by the fast Fourier transform (FFT). However, the FFT is inadequate for identifying non-stationary transient information because it has no time resolution [50]. In this context, an alternative approach is to segment the sequence into a set of short subsequences, with each subsequence centered on uniform time intervals and its DFT calculated separately, thereby obtaining the short-time Fourier transform (STFT) [51]. Thus, the STFT is defined by Kim et al. [52]

(1) $$STFT\;\left(t,\omega \right)=\;\overset{\infty }{\underset{-\infty }{\int }}h\left(u\right)f\left(t+u\right)e^{-j\omega u}\;du$$

where f(t) is a given signal in the time domain, t is the time, ω the frequency, and h(u) the temporal window function such as rectangular, Gaussian, Blackman, Hanning, Hamming, Kaiser, etc.

The result obtained by the STFT is a two-dimensional representation of the signal in time and frequency. However, the limitation imposed by Heisenberg’s uncertainty principle requires a relation between the resolutions (time–frequency). The accuracy in time and frequency are mainly determined through the window length, which is constant for all frequencies. In this way, a longer window results in a better resolution in the frequency domain. However, to obtain a more accurate time resolution, a window of smaller length is used. Therefore, it is necessary to know the resolution in time and frequency to obtain all relevant information in both domains [53]. The resolution in time and frequency can be defined by Equations (2) and (3), respectively.

(2) $$\Delta t=N*T_{s}$$

(3) $$\Delta F=m\frac{F_{s}}{N}$$

where N is the window length; $T_{s}$ the sampling period; $F_{s}$ the sampling frequency; and m the window coefficient, and where the sampling period is equal to the inverse of the sampling frequency and the window coefficient depends on the type of window used, e.g., m = 2 for a rectangular window.

Many statistical parameters are applied to the signals collected during the monitoring of manufacturing processes. The most-used statistic is the root mean square (RMS) [54], however, other statistics are also applied to these signals and are able to diagnose events that occur in the monitored processes. In this context, the application of counts [7], DPO (power deviation) [55], and ROP [56] is highlighted.

According to Lin et al. [57], the ratio of power (ROP) is a statistical method for analyzing the ratio of a given frequency band with the total power spectrum. The ROP statistic can be defined as

(4) $$ROP=\;\frac{\sum_{k=n1}^{n2}{\left|x_{k}\right|}^{2}}{\sum_{k=0}^{N-1}{\left|x_{k}\right|}^{2}}$$

where N is the block data size; n1 and n2 define the frequency range for the analysis; and $x_{k}$ is the *k*th discrete Fourier transform [58].

The study of similarity between two or more sensor signals, statistics, or parameters is of vital importance in the validation of new techniques. The correlation study between a widely known variable in relation to a new variable in the test phase is done through several statistics and indices. In this sense, the application of correlation coefficient [59], wavelet coherence [60], and magnitude-squared coherence [61] is highlighted

According to Scannell et al. [62], the magnitude-squared coherence (MSC) lists common frequencies between two signals and evaluates their similarity. The MSC between the time domain signals *x* and *y* can be calculated by Equation (5). Results in the interval between 0 and 1 indicate the level of spectral similarity between both time-domain signals.

(5) $$C_{xy}=\;\frac{{\left|P_{xy}\left(f\right)\right|}^{2}}{P_{xx}\left(f\right)P_{yy}\left(f\right)}$$

where $P_{xx}\left(f\right)$ and $P_{yy}\left(f\right)$ are the power spectral densities of *x* and *y* and $P_{xy}\left(f\right)$ is the cross power spectral density at frequency *f*.

## 5. Materials and Methods

### 5.1. Experimental Setup

Seven ceramic alumina (Al_2_O_3_) workpieces with 35 mm length × 8 mm width × 20 mm height and a Vickers microhardness of 1339 ± 47 HV1 (JIS R1610-1991 standard) were machined in a surface grinding machine, model RAPH 1055, from Sulmecanica. A resin-bond diamond grinding wheel, Dinser SD126MN50B2, with 15.5 mm thickness × 350 mm diameter was used in the tests. A single grinding pass was performed in the surface of each workpiece at a different depth of cut. Seven depth of cut values were tested, representing slight, moderate, and severe grinding conditions. Prior to the first test, the grinding wheel was dressed by a conglomerate-type dresser in order to ensure its best cutting performance. Coolant fluid was used in all tests. The workpieces were attached in the middle of a metal workpiece holder of 100 mm length × 35 mm width × 60 mm height by screws, which were adjusted with a torque wrench. The grinding parameters are shown in Table 1.

### 5.2. Workpiece Surface Assessment

After grinding, the subsurface of each workpiece was evaluated by means of a three-dimensional (3D) LEICA digital confocal microscopy (DCM) with 800× magnification. In addition to the confocal analysis, the surface quality assessment was complemented with surface roughness (R_a_) measurements, which were measured using a portable roughness tester Taylor Hobson 3+. A cut-off of 0.25 mm and a sampling length of 0.8 mm were considered. Each piece was divided into five equidistant points; three roughness measurements were taken from each point. The mean roughness was calculated for each workpiece, thus allowing the evaluation of the quality of the surface.

### 5.3. Data Acquisition

A low-cost piezoelectric diaphragm (PZT), model 7BB–35–3, from Murata Electronics, was attached to the workpiece holder with a cyanoacrylate of medium viscosity, from Tekbond. As in Viera et al. [29], the glue was evenly distributed over the entire diaphragm surface, thus maintaining a thickness of less than 1 mm. The piezoelectric diaphragm consists of (1) a bottom electrode (brass) of 35 mm diameter × 0.3 mm thickness (which is glued to the holder); (2) a piezoelectric ceramic of 25 mm diameter × 0.23 mm thickness (active element), and 3) an upper electrode (silver) of 23 mm diameter.

The study conducted by Freitas and Baptista [18] showed that the Murata piezoelectric diaphragms have a similar frequency response, determining that for most applications, the size of the diaphragm does not influence the results. Thus, the 35 mm model was chosen to validate the proposed approach. Other diaphragm models with different sizes and thicknesses can be tested in future studies. The piezoelectric diaphragm was glued to the center of the workpiece holder, thus positioning it as close as possible to the acoustic source (workpiece surface). Finally, the PZT diaphragm was covered with silicone in order to protect it from the coolant fluid. The PZT transducer was exclusively used in the passive configuration operating under *d*_31_ mode; in other words, the transducer was not excited by external sources; its function was to convert the acoustic waves generated during the process into electric voltage through the piezoelectric effect.

For comparison, an acoustic emission (AE) sensor was also fastened to the workpiece holder by a screw. The sensor was connected to a signal unit, model DM – 42, from Sensis manufacturer, which has an input–output gain of 3. The AE sensor consists of a metal housing of 30 mm × 20 mm × 20 mm in length, width, and height, respectively. Inside the metal housing is the piezoelectric ceramic (active element), as well as a filter and backing material, which reduces the resonance of the sensor. The AE sensor was screwed at a distance of 25 mm from the right end of the holder and at the same height as the PZT diaphragm.

**Figure 2:**
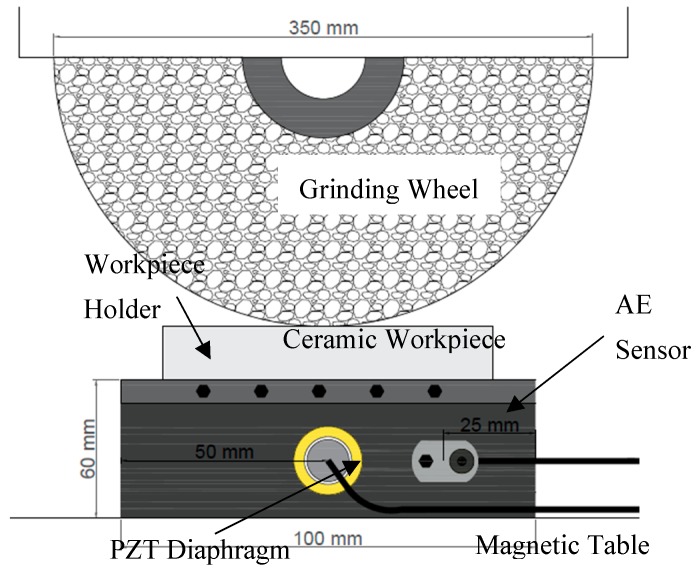
Experimental setup.

The level of saturation and sensitivity of the two sensors was verified before the grinding tests. Both sensors were attached to the workpiece holder in order to reproduce an industrial application, where changes in machine cycles would be unacceptable. An oscilloscope, model DL850, manufactured by Yokogawa, collected the AE and PZT raw signals at a sample rate of 2 MS/s. The PZT output was directly connected to the oscilloscope. On the other hand, the AE output was first connected to the signal unit, which improves the signal conditions. The aliasing effect was prevented by an anti-aliasing filter, inbuilt in the oscilloscope data acquisition board, with a 65-dB cutoff centered at 80% of the sampling frequency. The experimental setup is shown in Figure 2.

### 5.4. Signal Processing

The signals collected from the grinding tests were processed in MATLAB software. According to Ribeiro et al. [27], Moia et al. [63], and Lopes et al. [7], the AE sensor used in the tests has a frequency response of up to 300 kHz. However, as verified in the previous study of the same tests [29], the sensor presented significant frequencies up to 250 kHz. As shown by Freitas et al. [18], the PZT sensor has an efficient response of up to 200 kHz; the signal observed after this frequency has attenuations that can lead to erroneous results.

Considering the operational frequency bands of the sensors (up to 200 kHz for the PZT transducer and up to 300 kHz for the AE sensor), a digital bandpass filter (2 kHz to 300 kHz) was applied in order to eliminate noise. As there is a noise caused by the mechanical characteristics of the process and the data acquisition system, a digital Butterworth 10 order filter was also applied. The frequency of 2 kHz was chosen due to the fact that low frequencies present more information regarding noises than from the process. The signals were resampled at a rate of 1 MS/s, reducing the amount of data and respecting Nyquist’s theorem. It is worth mentioning that the resample function of MATLAB applies an anti-aliasing low-pass FIR filter to the signals and compensates for the delay introduced by the filter. The STFT of each grinding test was computed with the following specifications: 5000-points rectangular window and 90% overlap. Thus, through Equations (2) and (3), the resolutions of 5 ms in time and 400 Hz in frequency were obtained.

Two frequency bands were chosen (one per sensor). The frequency bands were chosen based on the absolute values of the STFT spectrograms. Thus, the selected frequency bands presented two characteristics: (1) Frequency intervals with similar values over time (on the same spectrogram) and (2) magnitude differences (color bar) related to the process conditions (between spectrograms). Subsequently, the ROP metric was calculated for each frequency band. The absolute STFT values and the time–frequency resolution allowed the calculation of the ROP metric in the time domain. It is worth mentioning that the ROP metric is traditionally calculated in the frequency domain, so its calculation in the time domain, proposed in this paper, expands its application in real-time monitoring systems.

The magnitude-squared coherence (MSC) was then calculated for three grinding tests: 25 μm (slight), 105 μm (moderate), and 350 μm (severe). In addition, the upper envelope of each result was obtained in order to present the MSC results more clearly; the envelope was calculated by dividing the MSC results into 2048-point intervals, which correspond to about 1 ms time intervals from the total grinding pass section. Finally, the correlation between the measured surface roughness and the ROP mean values for both sensors was obtained by a linear regression. Signals were normalized in order to eliminate amplitude differences caused by the sensitivity of each sensor.

## 6. Results and Discussion

**Figure 3:**
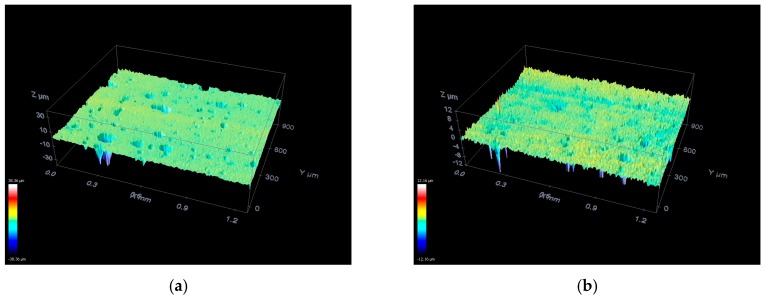

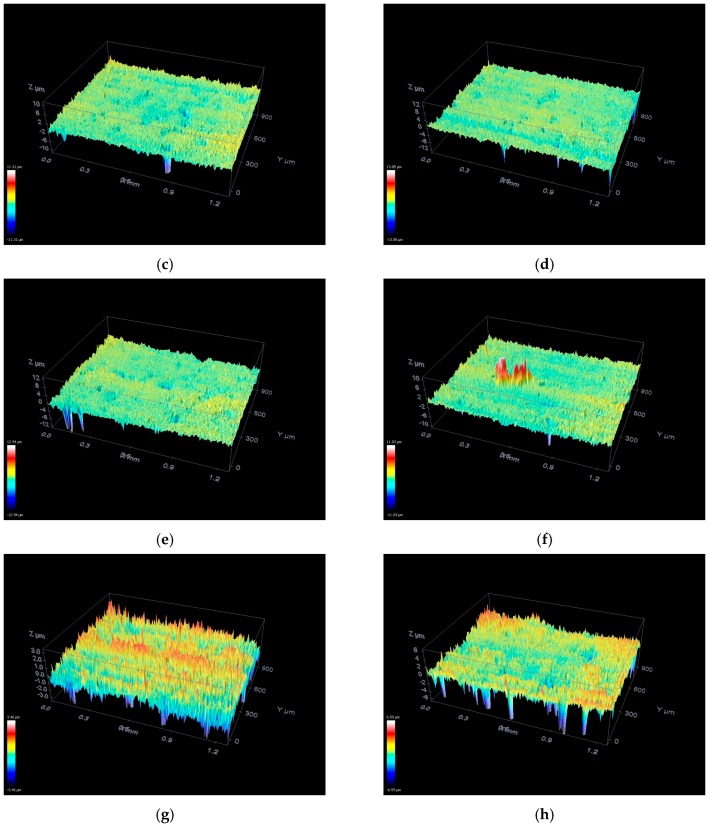
Confocal microscopy: (**a**) Without cutting; (**b**) 25 µm; (**c**) 35 µm; (**d**) 50 µm; (**e**) 105 µm; (**f**) 150 µm; (**g**) 210 µm; and (**h**) 350 µm.

**Figure 4:**
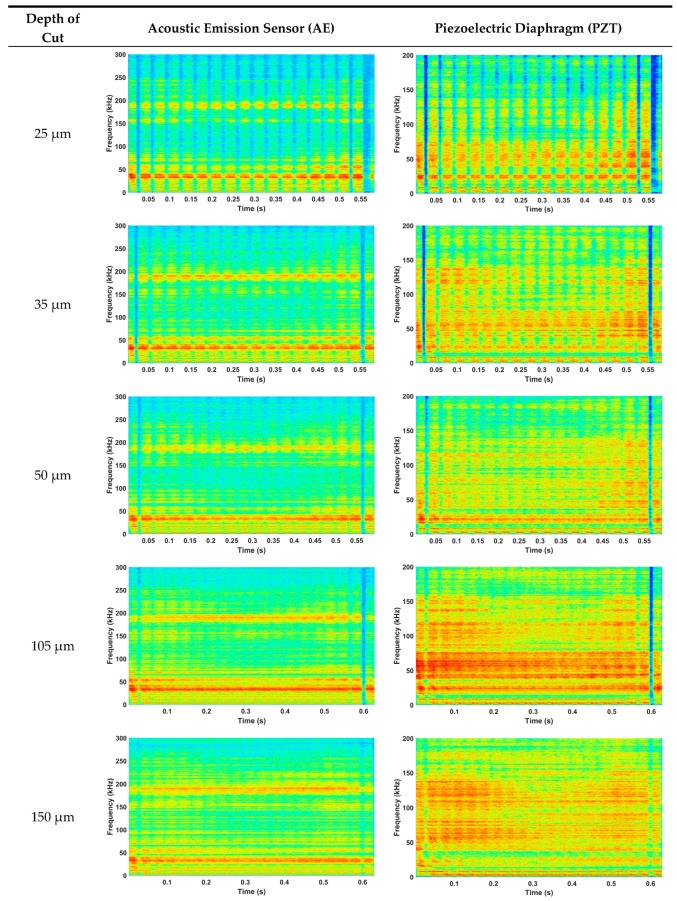

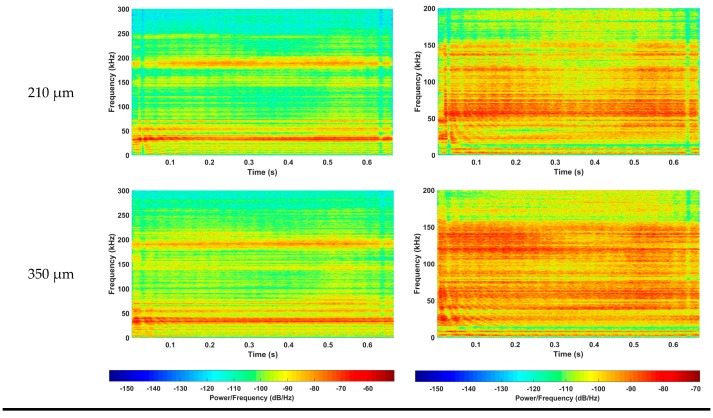
Short-time Fourier transform (STFT) spectrograms.

**Figure 5:**
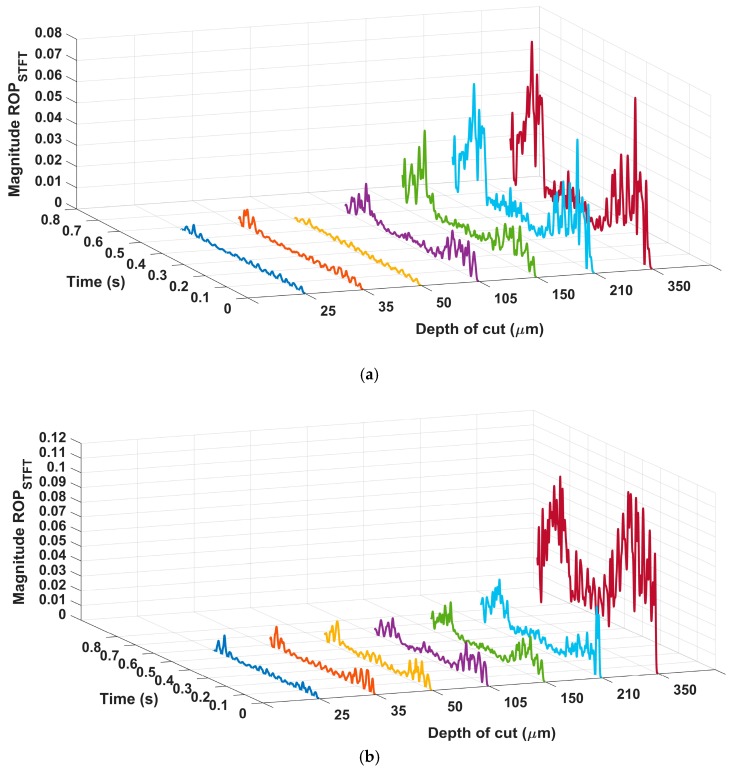
The ratio of power (ROP) of the (**a**) acoustic emission sensor (AE) and (**b**) piezoelectric diaphragm (PZT).

**Figure 6:**
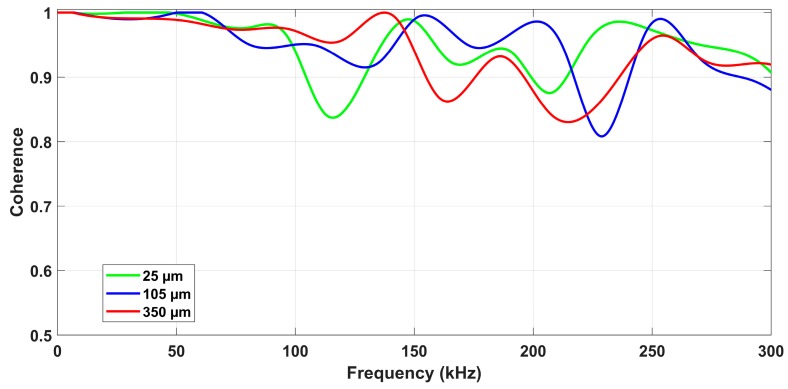
Magnitude-squared coherence between the AE sensor and PZT diaphragm at three grinding conditions.

**Figure 7:**
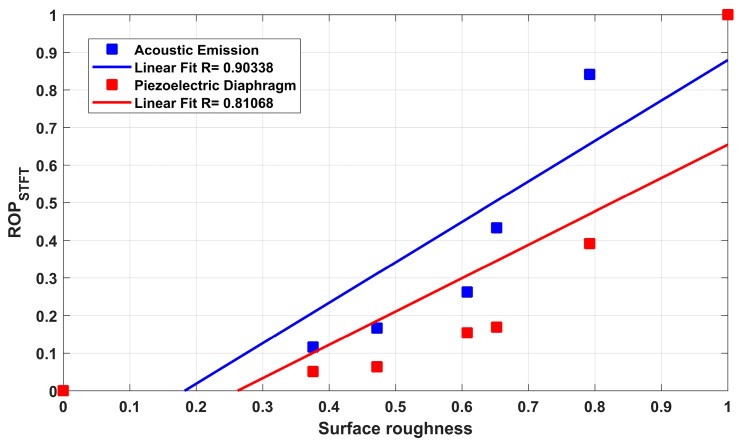
Correlation between ROP and surface roughness for both sensors.

This section presents the results obtained from the characterization of the ground ceramic workpieces and the digital processing of the signals from both sensors. The results of the subsurface assessment by confocal microscopy are shown in Figure 3. The mean surface roughness values (*R_a_*) measured with the portable roughness tester are presented in Table 2. Figure 4 presents the time–frequency analysis by STFT for both sensors and the seven cutting conditions. After the selection of frequency bands, the ROP parameter is shown in Figure 5. Figure 6 presents the magnitude coherence between the AE sensor and the PZT diaphragm at three cutting conditions. Finally, Figure 7 shows the correlation between the ROP metric and the surface roughness. All results were correlated with the surface quality of the ground workpieces.

### 6.1. Workpiece Surface Assessment

In order to detect micro-defects on the surfaces, confocal measurements were performed in a central region of each workpiece, as shown in Figure 3. As a reference, Figure 3a shows the microscopy of a workpiece prior to the grinding process (without cutting); a uniform surface can be observed with small imperfections caused by previous machining processes and short peak-to-valley distance. It can be seen in Figure 3 that the slight cutting conditions (Figure 3b–d) did not show any significant defects caused by the grinding process. However, some irregularities in the ceramic surface, such as cracks and porosity, can be observed, which resulted from previous machining processes.

The influence of the grinding process on the workpiece surface increases with the depth of cut. A bigger contact area between the workpiece surface and the abrasive grains of the grinding wheel generates an increased volume of material removed, which deteriorates surface quality and increases the number of surface cracks. In the moderate cutting conditions (Figure 3e,f), there is an increase of irregularities in the surface of the workpiece due to the increase of the cutting forces, which causes an increase in the grinding wheel wear and affects the surface quality of the workpiece. Figure 3f shows a predominant irregularity in the central-left part of the workpiece; this was caused by a crack that originated during the workpiece manufacturing process, which was enlarged by the grinding process. The other regions of the workpiece presented uniform irregularities, which are consistent with the severity of the process. The most significant effects on the workpiece surface were observed on the severe cutting conditions (Figure 3g,h); the irregularities and color differences in the images are related to the adjacency of the formed valleys and the distance between the peaks and valleys on the ground ceramic surface. The grinding effects mentioned above in the slight and moderate processes become more severe in the last two depth of cuts, where color changes can be observed throughout the workpiece surface. An increase in the peak-to-valley distance can be clearly observed, which represents a greater surface roughness.

The results of the mean surface roughness, measured with the portable roughness tester, are shown in Table 2. The results presented are the total mean of all measurements performed over the five regions that the workpiece surface was divided. As expected, a severe grinding process results in a higher mean roughness. The results presented in Figure 3 agree with those obtained in Table 2; it can be observed that the roughness values followed the same trend, rising directly with the increase of the depth of cut. According to Marinescu et al. [64], the maximum acceptable roughness value is of 1.6 μm for the steel grinding process. On the other hand, in the ceramic grinding process, a maximum roughness value of 1.0 μm is expected. Table 2 shows an increasing trend, with the lowest surface roughness value at 25 µm and the highest value at 350 µm. All roughness values are in accordance with the theory (less than 1.0 μm), which confirms that the ceramic grinding tests were successful. In addition, high standard deviation values are common for roughness measurements on ceramic components due to variations in peaks and valleys resulting from machining; these values increased as the cutting condition becomes more severe.

### 6.2. Signal Processing

The STFT spectrograms of both sensors for the three grinding conditions are shown in Figure 4. The color scale in the spectrograms represents the magnitude of a frequency in a given interval of time, the characteristic frequencies of the process, as well as the most influential frequencies, are shown in different shades of red colors. On the other hand, frequencies that are slightly sensitive to the grinding process are presented in shades of blue. The green, yellow, and orange colors, located in the middle of the color scale, represent frequencies sensitive to the process, but with a minor influence compared to the red ones.

In the spectrograms corresponding to the three slight grinding conditions (25 μm, 35 μm, and 50 μm), variations of magnitude can be observed over time. These variations were caused by the contact between the workpiece and the grinding wheel abrasive grains, which did not show a constant contact behavior, since many of the grains did not remove material from the workpiece surface, generating low levels of AE activity. Thus, little energy was released during the process, justifying the low harmonic content of the signals.

The two moderate grinding conditions (105 μm and 150 μm) showed higher magnitudes when compared to the slight conditions. An increase in red tones can be observed throughout the frequency spectrum, which is primarily caused by the grinding wheel wear and the process severity. The color pattern was shown to be more uniform due to the increased contact between the abrasive grains of the grinding wheel and the workpiece surface. The spectrograms representing the severe grinding processes (210 μm and 350 μm) presented uniform harmonic content over time. This behavior was caused by the continuous and severe contact between the grinding wheel grains and the ceramic component, generating more AE activity. In the three grinding conditions, a characteristic frequency was observed, with an approximate value of 35 kHz, present throughout the machining time. However, the more severe grinding condition presented higher harmonic content, assuming higher magnitude levels. The results obtained in Figure 4 are in agreement with the results of the direct measurements of Figure 3 and Table 2. As in Figure 3, it is possible to observe the color changes as the process severity increases, which is directly related to the surface roughness and surface quality of the ground workpiece. The attenuation of the PZT diaphragm response, near its maximum response region (200 kHz), can be clearly observed in the spectrograms. There is a frequency component close to 200 kHz, which can be easily observed in the AE spectrograms, whereas in the PZT spectrograms it is difficult to observe, especially in slight cutting conditions, which generate lower acoustic levels. For this reason, it is not recommended to choose frequency bands near the maximum response region of the sensors.

The differences between the spectrograms of both sensors are related to the characteristics of the sensors, such as: The frequency response of the piezoelectric diaphragm (200 kHz) [18] and the AE sensor (300 kHz) [7,27,63], the construction of the sensors (AE sensor housing), and the signal unit. Through the spectrograms generated by the STFT, it is possible to differentiate the cutting conditions of the grinding process. In the results of Figure 4, it was possible to observe the changes in magnitude through the different cutting conditions and continuity through the grinding time. The two sensors presented similar results, showing a good sensitivity to the acoustic activity generated by the ceramic grinding process. The low-cost PZT diaphragm presented stronger color shades, which represents a greater sensitivity to the process stimuli. However, the AE sensor has a higher frequency response band and a signal unit, which filters and establishes gains for the raw signals, which makes the signal more reliable and accurate over time.

Frequency bands were chosen by means of the spectrograms shown in Figure 4. The selection criterion was based on the continuity of the frequency band over time and on the color difference between each grinding condition. As a result, two frequency bands were chosen, one for each sensor. Based on the selection criterion, the (142–147) kHz band was chosen for the piezoelectric diaphragm, while for the AE sensor, the (138–143) kHz band was selected. The frequency bands, chosen directly from the matrices containing the absolute values of each STFT, were used to calculate the ROP metric, which presented characteristics related to the process conditions.

The ROP results, applied in the selected frequency bands for both sensors, are shown in Figure 5. A higher ROP level can be clearly observed as the depth of cut increases. The results of the three slight cutting conditions presented low magnitudes, while moderate and severe conditions showed a greater magnitude and peak-to-valley distance. This behavior results from the accumulation of chip in the cutting region, which generates higher cutting force, AE activity, and surface roughness. Thus, the grinding of ceramic components at a depth of cut of 350 μm generated the highest levels of ROP, STFT, and R_a_.

An increase in the ROP level at the beginning and at the end of the machining process can be clearly seen in Figure 5; all the cutting conditions showed the same characteristic. This is a typical behavior of the ceramic grinding process as it represents the first and last contact of the grinding wheel grains with the workpiece surface. The contact grinding area is smaller at the beginning and at the end of the machining process, regions in which the workpiece presents higher mechanical stress and a smaller area of energy propagation. This characteristic can be observed in Figure 2, from 0.0 to 0.2 and 1.0 to 1.2 on the *x*-axis, with color changes representing slight increases in surface roughness. In the middle region of the workpieces, the surface roughness presented more uniform values; these changes become clearer in the depths of cut that represent severe cutting conditions.

### 6.3. Correlation Analysis

The results of the magnitude-squared coherence (MSC) for three grinding conditions are observed in Figure 6. The coherence was obtained from the raw acoustic emission and piezoelectric time-domain signals. Each of the MSC values indicates how well the PZT signal corresponds to the AE signal for each frequency, with 1 representing an ideal coherence parameter and 0 representing the complete lack of relation between the signals. It can be observed that even in the severe cutting condition, where higher acoustic activity and spectral content is expected, the MSC values were higher than 80%. The successful results presented in Figure 6, directly related to the signals of both sensors under various grinding conditions, reinforce the application of the low-cost piezoelectric diaphragm in the monitoring of the grinding process of advanced ceramics. The lower values of MSC were observed in the frequency range between 200 and 300 kHz; this is due to the attenuation of the PZT sensor, which increases the difference between the signals. The low-cost PZT diaphragm demonstrated a similar response to the AE sensor, being sensitive to the stimulus caused by the grinding processes tested in this test bench.

A linear regression was performed and the coefficient of determination (R) was calculated in order to show the similarity between the measured surface roughness and the signals of each sensor, as shown in Figure 7. The coefficient of determination was 0.90338 for the AE sensor and 0.81068 for the PZT diaphragm. Although the linear fit was better for the AE sensor, there is a high coefficient for both sensors, which is also confirmed by the coherence of Figure 6. Thus, the ROP metric can contribute to the evaluation of the surface quality of the ground ceramic components, as indicated in the results obtained by the linear regression. The linear fit was close to 45°, i.e., R very close to 1, indicating a high correlation between the signals and the measured roughness, which is the focus of this study. As expected, the AE sensor showed a better response to the acoustic activity generated during the grinding process, which is related to the surface quality of the workpiece, because of the metal housing, filter, and signal unit, which improve the signal conditions. However, the signals from the PZT diaphragm, studied in this work, showed a high degree of correlation (greater than 80%), confirming the feasibility of using the low-cost PZT diaphragm in the monitoring of the surface quality of ground workpieces through the acoustic activity generated during the process. The PZT diaphragm was able to detect the same changes in acoustic activity as the AE sensor.

## 7. Conclusions

In this work, a flexible and low-cost piezoelectric diaphragm was applied for surface quality monitoring in grinding of ceramic materials. The PZT diaphragm was also compared to an AE sensor, which is widely used for monitoring in several machining fields. In addition, a new time-domain ROP metric, based on the short-time Fourier transform, was proposed with the objective of estimating the surface quality of the workpieces. Therefore, the following conclusions can be drawn from this work:

- A grinding process performed at a high depth of cut results in increased mean surface roughness and acoustic activity levels;
- Machining under severe conditions caused greater irregularities in the ceramic surfaces;
- The increase in ROP values was directly related to the increase in surface roughness, which was caused by the increase in process severity;
- The PZT diaphragm responded satisfactorily to the process stimuli; the results were supported by the behavior of the AE sensor;
- The coherence analysis between the responses of the low-cost PZT diaphragm and the AE sensor reinforced the results obtained, proving the viability of using the PZT diaphragm in the monitoring of the grinding process; all coherence values were higher than 80%;
- The results demonstrated the feasibility of applying the low-cost PZT diaphragms for the tested machining conditions and can be extended to other low-cost sensors and materials.

The use of the PZT diaphragm in the monitoring of the surface quality of ground ceramic components, together with time–frequency domain analysis techniques, is new, and there are several possibilities for future applications and studies. Future work can be performed with new signal analysis methods and grinding parameters. The use of neural networks of classification or even estimation can be studied further.

## Figures and Tables

**Table 1:** Grinding parameters.

**Grinding Speed**	
Cutting speed ($v_{s}$)	33 m/s
Worktable speed ($v_{w}$)	58 mm/s
Depth of cut (µm)	25–35–50–105–150–210–350
**Lubri-Cooling Specification**	
Fluid	Shell–DMS 3200 F-1
Flow rate	27.5 L/min
Pressure	<0.7 MPa
Concentration	4% oil-water

**Table 2:** Mean surface roughness (Ra) measured with a portable roughness tester.

Depth of Cut (a-µm)	Surface Roughness (R_a_-µm)
25	0.516 ± 0.027
35	0.620 ± 0.033
50	0.647 ± 0.037
105	0.684 ± 0.040
150	0.697 ± 0.042
210	0.736 ± 0.051
350	0.793 ± 0.055
