# Erratum: Aulestia Viera, M., et al. A Time–Frequency Acoustic Emission-Based Technique to Assess Workpiece Surface Quality in Ceramic Grinding with PZT Transducer. *Sensors* 2019, *19*, 3913

**DOI:** 10.3390/s20082387

**Published:** 2020-04-22

**Authors:** Martin A. Aulestia Viera, Paulo R. Aguiar, Pedro Oliveira Junior, Felipe A. Alexandre, Wenderson N. Lopes, Eduardo C. Bianchi, Rosemar Batista da Silva, Doriana D’addona, Andre Andreoli

**Affiliations:** 1 Department of Electrical Engineering, São Paulo State University—UNESP, Av. Eng. Luiz Edmundo Carrijo Coube, 14-01, Bauru 17033-360, Brazil; paulo.aguiar@unesp.br (P.R.A.); pedrojunior5@aedu.com (P.O.J.); felipe.alexandre@unesp.br (F.A.A.); wenderson.nascimento@unesp.br (W.N.L.); andreoli@feb.unesp.br (A.A.); 2 Department of Mechanical Engineering, São Paulo State University—UNESP, Av. Eng. Luiz Edmundo Carrijo Coube, 14-01, Bauru 17033-360, Brazil; bianchi@feb.unesp.br; 3 School of Mechanical Engineering, Federal University of Uberlandia, Av. João Naves de Avila 2121, Uberlandia 38408-100, Brazil; rosemar.silva@ufu.br; 4 Fraunhofer Joint Laboratory of Excellence on Advanced Production Technology (Fh-J_LEAPT Naples) Department of Chemical, Material and Industrial Production Engineering, University of Naples Federico II, Piazzale Tecchio 80, 80125 Naples, Italy; daddona@unina.it

The authors wish to make the following erratum to this paper [1].

Funding and Acknowledgement are incorrect and must be replaced by the following Funding and Acknowledgments:

**Funding:** This research was funded by the São Paulo Research Foundation (FAPESP), grant number #2018/07292-0, Coordination of Superior Level Staff Improvement (CAPES) and National Council for Scientific and Technological Development (CNPQ), under grant 306435/2017-9 for supporting this research work.

**Acknowledgments:** The authors are grateful to the São Paulo Research Foundation–FAPESP, Coordination of Superior Level Staff Improvement—CAPES and National Council for Scientific and Technological Development—CNPQ for financial support.

The authors would like to apologize for any inconvenience caused to the readers by these changes.
